# Synthesis and Evaluation of a Rationally Designed Click-Based Library for G-Quadruplex Selective DNA Photocleavage

**DOI:** 10.3390/molecules200916446

**Published:** 2015-09-10

**Authors:** Dominic McBrayer, Sean M. Kerwin

**Affiliations:** 1 Department of Chemistry and Biochemistry, The University of Texas, Austin, TX 78712, USA; E-Mail: dmcbrayer@cm.utexas.edu; 2 Division of Medicinal Chemistry, College of Pharmacy, The University of Texas, Austin, TX 78712, USA

**Keywords:** DNA, photocleavage, alkyne-azide click reaction, PAGE

## Abstract

DNA containing repeating G-rich sequences can adopt higher-order structures known as G-quadruplexes (G4). These structures are believed to form within telomeres and the promoter regions of some genes, particularly in a number of proto-oncogenes, where they may play a role in regulating transcription. Alternatively, G4 DNA may act as a barrier to replication. To investigate these potential biological roles, probes that combine highly selective G4 DNA targeting with photocleavage activity can allow temporal detection of G4 DNA, providing opportunities to obtain novel insights about the biological roles of G4 DNA. We have designed, synthesized, and screened a small library of potential selective G-quadruplex DNA photocleavage agents incorporating the G-quadruplex targeting moiety of 360A with known photocleavage groups linked via “click” chemistry.

## 1. Introduction

G4 DNA is highly stable structural conformation that is composed of a series of stacked tetrads composed of four guanines joined through Hoogsteen hydrogen bonds. Monovalent cations such as potassium or sodium occupy the central channel formed by these stacked tetrads. G4 DNA may be formed either by the joining of several strands of single-stranded DNA or intramolecularly within a single-stranded DNA molecule. There are many different G4 conformations that can be adopted, based on the location of loops and the directionality of the strand(s). These conformations are highly dependent on the sequence of the DNA as well as the type of monovalent cation present [1]. Compounds that incorporate planar aromatic systems and positive charges have been shown to bind to G4 DNA with varying specificities, most often by stacking on the planar ends of the G4 DNA or, less commonly, through association with the loops [2].

While the formation of G4 structures *in vitro* has been known for decades [3], direct evidence for their appearance and roles in biological systems has been primarily limited to single celled organisms such as ciliates [4] and bacteria [5]. However, there is a significant amount of indirect evidence in support of biologically relevant roles in multicellular organisms, including humans. Helicases that have been shown to resolve G4 structures faster and more selectively than they do duplex or other DNA secondary structures suggest that G4 structures do form *in vivo* and need to be resolved, likely because they pose barriers to replication and transcription. Mutations in some of these helicases are associated with disease states such as Bloom’s Syndrome and Werner’s Syndrome [6]. Further, sequences that lend themselves to G4 formation are more prominent in many (including human) genomes than would be expected to be present due to chance, suggesting selection for such sequences [7]. These sequences are also disproportionally associated with the promoters of proto-oncogenes [8], suggesting roles in gene regulation and making them interesting potential targets for anti-cancer therapy. The ability of the human telomeric DNA sequence to form G4 structures has also made it one of the most targeted sequences for potential cancer treatments [9].

In order to better understand the biological roles of G4 structures, tools are needed that are capable of directly detecting them within biologically relevant contexts. Small molecules capable of binding selectively to G4 DNA, and which incorporate an inducible sequence are promising candidates to function as such tools. There have been several compounds developed that show reasonable binding selectivity to G4 DNA [10,11]. These can serve as a framework for the development of not only potential drugs, but also for the development of G4-selective probes.

In this study, we developed a library designed around the principle of combining a “targeting” scaffold moiety based on an established G4 DNA ligand with various “warhead” photocleavage moieties. This strategy has given insight into the structure-activity relationship (SAR) associated with G4 DNA photocleavage ligands that will facilitate the design of other small molecular G4 DNA probes.

## 2. Results and Discussion

### 2.1. Library Design

**Figure 1:**
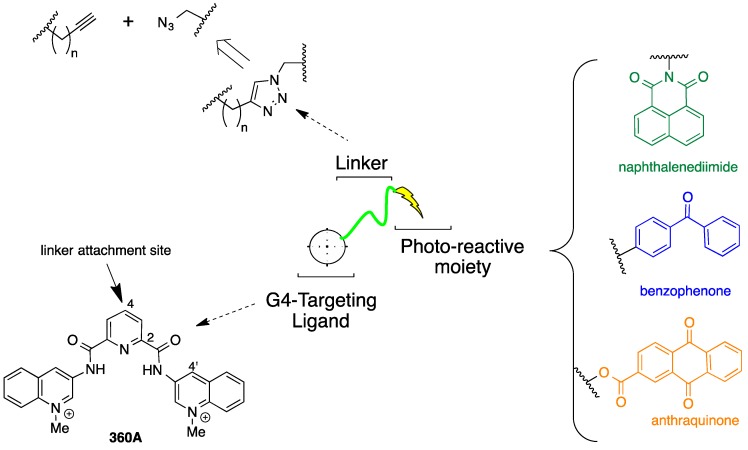
Design of a library of potential G-quadruplex DNA photocleavage agents.

The strategy that was employed to design a library of potential G-quadruplex photocleavage agents is shown in Figure 1. A G-quadruplex DNA targeting moiety is linked via variable-length linkers to a variety of established DNA photoreactive groups. The known G-quadruplex binding di(quinolin-3-yl)pyridine-2,6-dicarboxamide 360A was selected as the G4-targeting ligand based upon its selectivity and the relative ease of incorporation of functionality at the core pyridine 4-position [12]. To facilitate library construction, the linker was designed to incorporate a triazole unit, which could be assembled from copper-catalyzed coupling of appropriate terminal alkynes with different azides [13]. In implementing this approach, the terminal alkyne partners are G-quadruplex targeting ligands appended with different length ω-alkynylalkyl chains and the azides are derived from different photoreactive groups. These photoreactive groups were selected to explore different potential DNA cleavage modes. Photoexcited benzophenones undergo very efficient intersystem crossing, leading to DNA cleavage pathways proceding exclusively vie the triplet excited state [14]. Naphthalenediimides are known to photocleave duplex DNA via both singlet oxygen generation and electron-transfer processes [15]. Anthraquinones affect DNA photocleavage by electron transfer [16].

### 2.2. Synthesis of a Click-Based Library of G-Quadruplex Photocleavage Agents

**Scheme 1:**
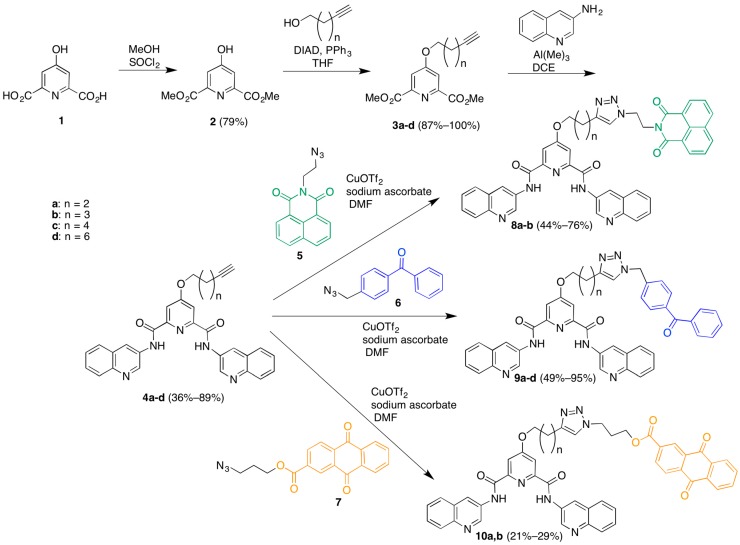
Synthesis of a library of triazole-linked G-quadruplex-photoreactive group molecules.

The synthesis of the library commenced with the preparation of a series of terminal-alkyne functionalized 360A derivatives (Scheme 1). Chelidamic acid monohydrate was converted to the known diester **2** [17] by reaction with thionyl chloride in methanol. The ester **7** was subjected to Mitsunobu coupling with a series of terminal alkyne alcohols to afford compounds **3a**–**d**, which differ from each other only by the length of the linker carbon chain. The diesters **3a**–**d** were treated with 3-aminoquinoline and trimethylaluminum in 1,2-dichloroethane (DCE) to afford the diamides **4a**–**d**. Next, these diamides were subjected to “click” reaction conditions in DMF with the different azide-functionalized photoactive moieties to afford three different series of compounds: the naphthalenediimides **8a**,**b**, the benzophenones **9a**–**d**, and the anthraquinones **10a**,**b**. DMF proved a more suitable solvent than aqueous *t*-BuOH [18] for these couplings, although it required the use of copper triflate as a copper source due to the low solubility of copper sulfate in the new solvent system, which was exacerbated by the need for stoichiometric copper. Stoichiometric copper was required due to the copper-chelating capacity of the pyridine-2,6-dicarboxamide [19,20] based scaffold. Addition of copper was met by an immediate color change of the solution to a deep green, and the triazole product could only be isolated when employing a slight excess (1.1 equivalents) of copper salt. We presume this is due to the rapid formation of a catalytically inactive 1:1 substrate-copper complex. This copper-chelating capacity also made purification and characterization of the product triazoles more difficult as the complexes needed to be disrupted in order to obtain free compound (see associated experimental). The additional steps required to disrupt the complex contribute to the modest yields for these coupling reactions.

**Scheme 2:**
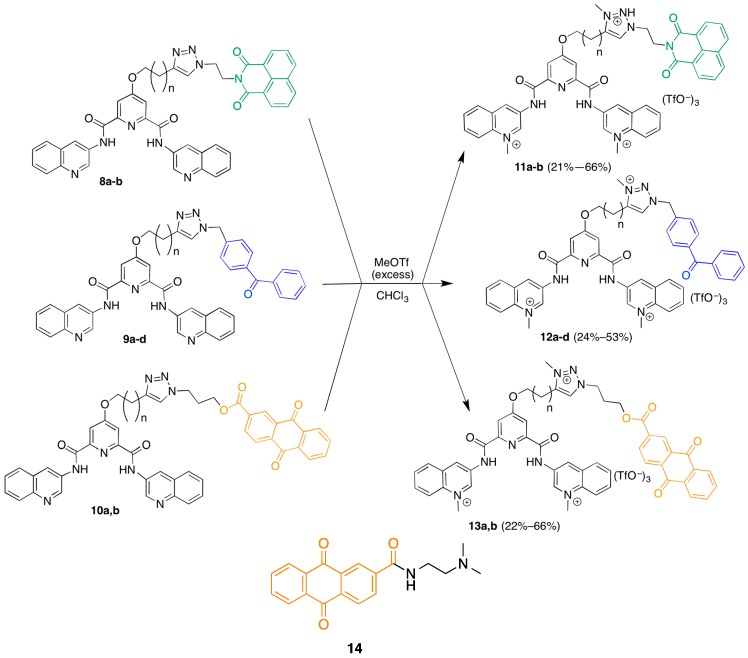
Preparation of potential G-quadruplex DNA photocleavage agent library.

Finally, methylation of the triazoles **8**–**10** in the presence of excess methyl triflate in chloroform gave the trimethylated products **11a**,**b**; **12a**–**d**; and **13a**,**b** (Scheme 2). The somewhat unexpected formation of trimethylated products was established by the presence of two different *N*-methyl resonances for these compounds in the ^1^H-NMR spectra and the observation of [M − OTf]^+^ ions in the MADLI MS and [M − 3TfO]^3+^ ions in the ESI MS. The location of these methyl groups was established indirectly through the following experiment. A small-scale methylation of **4a** with methyl triflate, followed by click reaction with azide **5** in water/*t*-BuOH afforded a crude dimethylated product that was characterized by ^1^H-NMR and LRMS. Comparison of the ^1^H-NMR chemical shifts of this product *vs.* those of the trimethylated **11a** showed substantial differences in the linker methylene peak positions best accounted for by methylation on the triazole ring in the case of **11a**. It should be noted that attempts to carry out this sequence of reactions on larger scale for the preparation of the dimethylated triazole compounds failed due to the very poor mass recovery from the final copper-catalyzed coupling reactions. This was due to the difficulties in isolating these water-soluble compounds, especially in the presence of the copper salts employed in the final coupling carried out in aqueous *t*-BuOH.

### 2.3. G-Quadruplex DNA Photocleavage

These library compounds were then tested for their ability to photocleave and bind to the F21T and cMYC G-quadruplexes. The photocleavage experiments were adapted from our group’s published photocleavage assay employing 6-carboxyfluorescein (FAM) and carboxytetramethylrhodamine (TAMRA) labeled G-quadruplex-forming oligonucleotides [21]. Two G-quadruplex-forming sequences were examined: F21T incorporated the human telomeric repeating sequence: 5′-FAM-dGGG(TTAGGG)_3_-TAMRA-3′, and F-c-MYC22m-T (cMYC) incorporated a modified sequence from the c-MYC gene promoter that has been shown to form a single G-quadruplex conformation *in vitro* [22]: 5′-FAM-dTGAGGGTGGGTAGGGTGGGTAG-TAMRA-3′.

Photocleavage reactions were carried out in potassium cacodylate buffer with 100 nM fluorescent-labeled oligonucleotides and 500 nM of library compound. After irradiation (UVA lamps) for 30 min, the DNA photoceavage products were either analyzed directly by PAGE or first treated with hot piperidine before PAGE analysis. 

**Figure 2:**
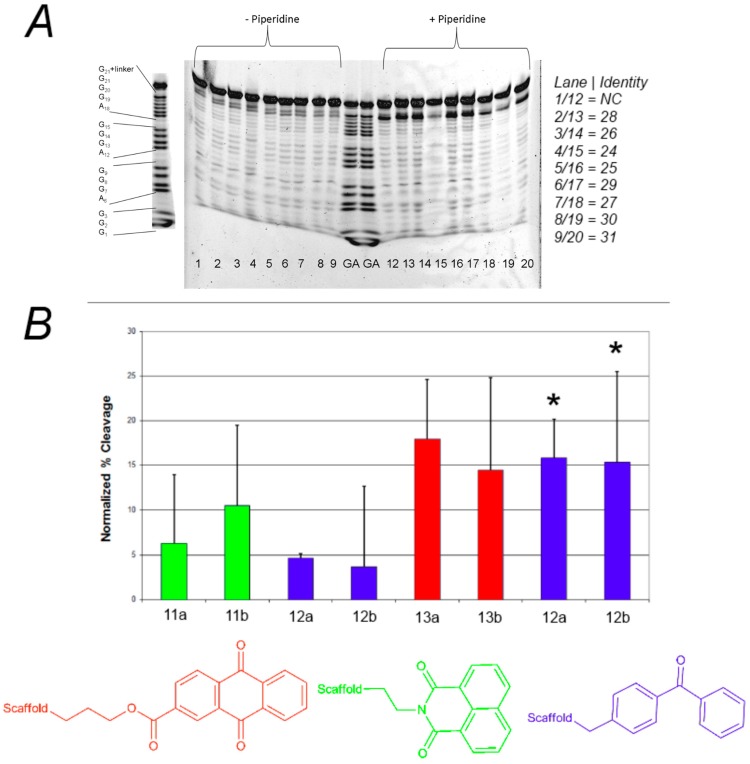
(**A**) Effect of photoreactive group on the photochemical cleavage of G-quadruplex DNA by click-based compound library members. (**A**) Example polyacrylamide gel of photocleavage reactions of F21T after 30 min UV irradiation in the presence of 500 nM click-based compound library members **11a**,**b**; **12a**–**d**; and **13a**,**b**; (**B**) Quantification of G-quadruplex photochemical cleavage from gel electrophoresis analysis after irradiation and piperidine/heat treatment. Unless indicated, F21T was employed as the G-quadruplex substrate. Red, green, and blue bars correspond to compounds incorporating anthraquinone, naphthalimide, and benzophenone respectively. * FcMycT photocleavage data for comparison.

As shown in Figure 2, none of these compounds were very effective as photocleavage agents, with the highest apparent cleavage levels around 25%–30% and most at 0%–15%. The photocleavage ability of these compounds was not increased by irradiation with shorter wavelength light (UVB lamps, data not shown). Furthermore, there was no clear pattern of preferred sites of photocleavage for any of the three different photoreactive groups (Figure 2A), indicating that the cleavage may be occurring through a diffusible intermediate. In general, the F-c-MYC22m-T G-quadruplex was more susceptible to photocleavage by these compounds when compared to F21T (Figure 2B, bars with asterisks).

**Figure 3:**
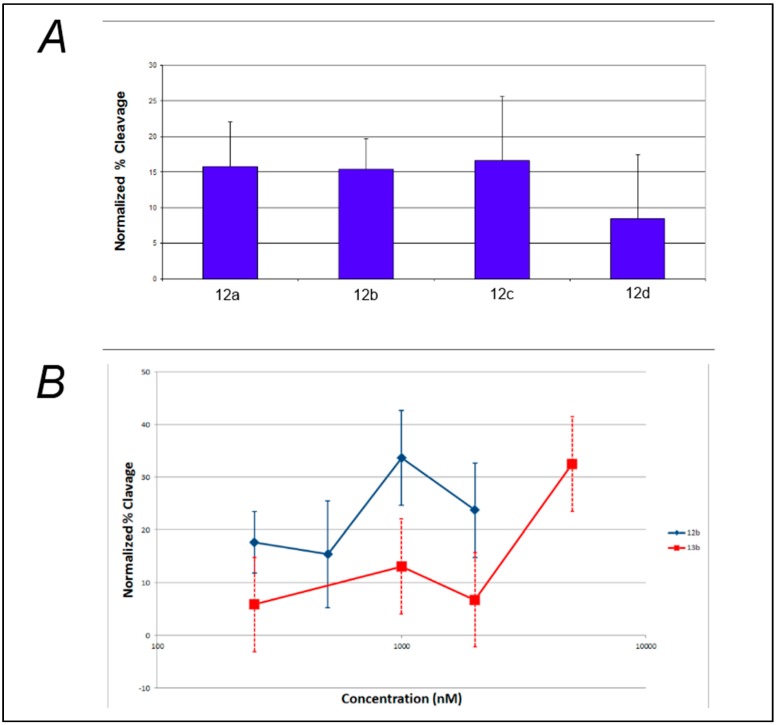
Effect of compound linker length and concentration on photochemical cleavage of G-quadruplex DNA. (**A**) Photochemical cleavage of FcMycT irradiated under UVA light in the presence of 500 mM of benzophenone-containing compounds of different linker lengths: **12a** (*n* = 2), **12b** (*n* = 3), 12c (*n* = 4) and **12d** (*n* = 6); (**B**) Photochemical cleavage of FcMycT irradiated in presence of different concentrations of benzophenone- (**12b**, blue line) and anthraquinone-containing (**13b**, red line) compounds of the same linker length (*n* = 3).

**Figure 4:**
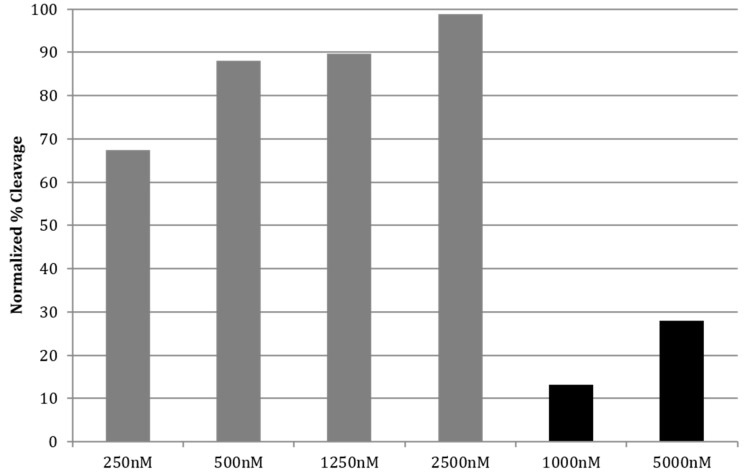
Photochemical cleavage of F21T by control compound **14** (black bars) compared with cleavage by TMPyP4 (gray bars) after 120 min of UVA irradiation.

Analysis of the effect of linker length on the efficiency of photocleavage of F-c-MYC22m-T by the benzophenone-containing compounds **12a**–**d** (Figure 3A) demonstrates little change in photocleavage with linker length. Furthermore, there was not a clear correlation between compound concentration and the extent of photocleavage of FcMycT for either the benzophenone-containing **12b** or the analogous anthraquinone compound with the same linker length **13b** (Figure 3B). This latter result was particularly surprising, given that some of the first examples of G-quadruplex DNA ligands were anthraquiones [23]. Therefore, to verify the ability of the anthraquinone moiety to cleave these G4 DNA structures as well as to investigate its binding, compound **14**, which incorporates the anthraquinone moiety functionalized through amidation to give a tertiary amine for solubility, was prepared and tested. Concentration-dependent cleavage was observed for **14**; however, at levels well below those of the well-established G-quadruplex DNA photocleavage agent TMPyP4 (Figure 4).

### 2.4. G-Quadruplex DNA Binding

**Figure 5:**
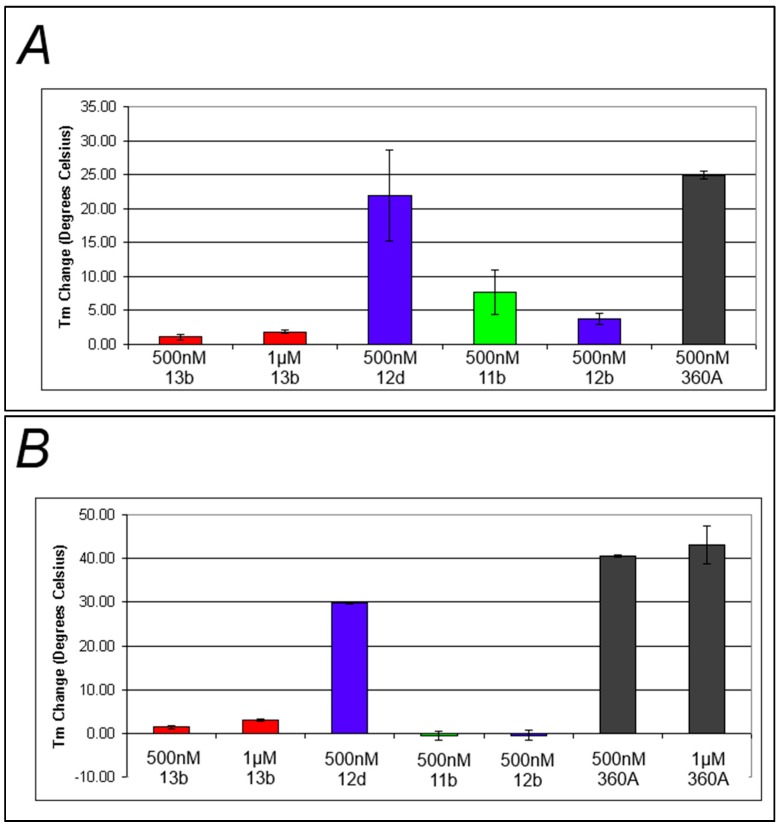
Changes in T_m_ upon formation of the DNA-compound complex (**A**) Average melt data for representative compounds incubated with FcMycT; (**B**) Average melt data for representative compounds incubated with F21T, Black, blue, green, and red bars represent positive control, benzophenone-incorporated, naphthalimide-incorporated, and anthraquinone-incorporated compounds respectively.

It was postulated that the presence of the triazolium moiety introduced in the final step of the synthesis might interfere with compound binding and/or photoactive group orientation, resulting in the lowered levels of photocleavage of these G-quadruplex DNA structures. In order to test this, the ability of a subset of the library compounds to bind to F21T and FcMycT was determined by FRET-based ΔT_m_ experiments. The degree of binding is assumed to be proportional to the increase of the T_m_ of the complex relative to the T_m_ of the untreated DNA [24]. Most of the compounds tested exhibited minimal binding as evidenced by ΔT_m_ shifts of only a few degrees Celsius, especially when compared to the large stabilization exhibited by **360A** (Figure 5). Interestingly, G-quadruplex DNA binding was partially restored in the case of **12d**, the benzophenone-containing compound with the longest linker length. This suggests that shorter linkers are not well tolerated for G-quadruplex DNA binding by these compounds, perhaps due to the positioning of the triazolium group. 

The results from the G-quadruplex photocleavage and binding studies reported here suggest several potential areas for improvement in the design of photoactive probes targeting G-quadruplex DNA. While longer linkers were stabilizing to the DNA-compound complex, clearly allowing binding, this did not appear to improve photocleavage performance. This may be because of the nature of the triazolium linker used. While increasing linker length would alleviate destabilization of the DNA-compound complex by increasing the distance between the primary DNA-binding moiety of the compound and the triazolium group, the distance between the triazolium group and the photoactive moiety remains unchanged. If the triazolium group is responsible for poor orientation of the photoactive group, then poor photocleavage performance would not necessarily be improved by increased linker lengths. 

## 3. Experimental Section 

### 3.1. General

The HPLC purified fluorescent dual-labeled oligonucleotides, F21T, (5′-FAM-dGGG(TTAGGG)3-TAM-3′), F-c-Myc22m-T, (5′-FAM-dTGAGGGTGGGTAGGGTGGGTAG-TAM-3′) and hairpin structure F-mixed-T, (5′-FAM-dGCATGCTTTTGCATGC-TAM-3′; FAM: 6-carboxyfluorescein; TAM: tetramethyl-rhodamine) were purchased from Integrated DNA Technologies and used without further purification. The dual-labeled oligonucleotides were diluted from water stock solution to 400 nM in 50 mM sodium or potassium cacodylate, pH 7.4 and annealed by heating to 95 °C for 5 min in a water bath, followed by slowly cooling to room temperature. FRET melting experiments confirmed G-quadruplex formation with melting temperatures of 48 °C, 56 °C and 85 °C (Na+F21T, K+F21T and F-c-Myc22m-T respectively) which correspond closely to the literature values for each G-quadruplex [24,25,26].

All reactions were carried out under argon in oven-dried glassware with magnetic stirring. Unless otherwise noted, all materials were obtained from commercial suppliers and were used without further purification. THF was distilled from sodium/benzophenone prior to use. Dichloromethane and 1,2-dichloroethane were distilled from CaH_2_ prior to use. Unless otherwise noted, organic extracts were dried with Na_2_SO_4_, filtered through a fritted glass funnel, and concentrated with a rotary evaporator (20–30 mmHg). R_f_ values are reported for analytical thin-layer chromatography (TLC) performed on EM Reagent 0.25 mm silica gel 60-F plates with UV light or KMnO_4_ visualization. Flash chromatography was performed with EM Reagent silica gel (230–400 mesh) using the mobile phase indicated. Melting points (open capillary) are uncorrected. Unless otherwise noted, ^1^H- and ^13^C-NMR spectra were determined in CDCl_3_ or *d*_6_-benzene on a spectrometer operating at 400 and 100 MHz, respectively, and are reported in ppm using solvent as internal standard (7.26 ppm for ^1^H and 77.0 ppm for ^13^C in CDCl_3_. 7.15 ppm for ^1^H and 128.0 ppm for ^13^C in *d*_6_-benzene). All mass spectra were obtained in the positive mode either by chemical ionization using methane as the ionizing gas or by electrospray ionization.

### 3.2. Library Synthesis

3-Azidopropyl 9,10-dioxo-9,10-dihydroanthracene-2-carboxylate (**7**). 100 mg (1 eq, 0.37 mmol) of 9,10-dioxo-9,10-dihydroanthracene-2-carbonyl chloride was dissolved in 3 mL freshly distilled dichloromethane in an oven-dried flask under argon. This flask was then cooled to 0 °C. In a separate oven-dried flask, 150 mg (2.55 eq, 0.92 mmol) of 62 wt % 3-azidopropan-1-ol [27] in DMF was dissolved in 1 mL of distilled dichloromethane under argon. 52 µL (1 eq, 0.37 mmol) of freshly distilled triethylamine was then added. The solution containing the alcohol and the amine was then added slowly under argon to the flask containing the acyl chloride at 0 °C. The reaction was then allowed to return to room temperature and was stirred for 19 h when it appeared complete by TLC (75% ethyl acetate in hexanes). After flash chromatography (25% ethyl acetate gradually up to 50% ethyl acetate in hexanes), 74 mg of the light yellow solid was obtained (approximately 80% purity, 45% yield). mp < 125 °C; IR (KBr) 3133.59, 2105.89, 1727.91, 1679.70, 1590.99, 1402.00, 1332.57, 1276.65, 1249.65, 1164.79, 1037.52, 939.16, 802.24, 703.89 cm^−1^; ^1^H-NMR (400 MHz, CDCl_3_) δ 8.9 (1H, s), 8.3–8.4 (4H, m), 7.80–7.83 (2H, m), 4.5 (2H, t, *J* = 6.0 Hz), 3.5 (2H, t, *J* = 6.6 Hz), 2.1 (2H, p, *J* = 6.4 Hz); ESIMS *m/z* 358.1 ([M + Na^+^]^+^, 100%); HRESIMS calc for C_18_H_13_N_3_NaO_4_^+^ 358.07983, found 358.07973.

Dimethyl 4-oxo-1,4-dihydropyridine-2,6-dicarboxylate (**2**) [28]. Distilled methanol that had been stored under argon over 4 Å molecular sieves (3.1 mL, 31 eq, 77.5 mmol) was added to a oven-dried flask under argon and the flask was then placed in an ice bath. Slowly, 1.1 mL (6.2 eq, 15.5 mmol) of thionyl chloride was added. The solution was allowed to stir for 5–10 min before 500 mg (1 eq, 2.5 mmol) of chelidamic acid was added under increased argon flow. The flask was outfitted with an oven-dried condenser. The mixture was then stirred for 72 h, under argon, allowing the ice bath to slowly warm to room temperature. The faintly yellow solution was then diluted approximately 2× with methanol, transferred to a larger flask, and the solvent removed under reduced pressure to give a white solid residue. The flask was then placed in an ice bath for 15 min before 3 mL of chilled distilled water was added with swirling, followed by 0.75 mL of chilled 10% sodium carbonate solution and 0.75 mL of chilled 50% aqueous methanol. After swirling, the mixture was allowed to stand in the ice bath for 20 min before being filtered under reduced pressure and washed with 3 mL, 3 mL, and 1 mL portions of chilled 50% aqueous methanol, giving 500 mg of crude white product. The crude product was adsorbed to 1 g of silica and purified through chromatography on a silica plug (about 5–6 g SiO_2_, EtOAc as eluent), giving 417 mg of purified product as a white solid (79% yield). ^1^H-NMR (400 MHz, CDCl_3_) δ 10.0 (1H, s), 7.5 (2H, s), 4.0 (6H, s) (matches lit. [21]); ^13^C-NMR (100 MHZ, CDCl_3_) δ 172.8, 163.3, 144.1, 117.7, 53.3 (matches lit. [21]).

Mitsunobu Coupling General Procedure: Dimethyl 4-(pent-4-yn-1-yloxy)pyridine-2,6-dicarboxylate (**3a**). 300 mg (1 eq, 1.42 mmol) of **7** was suspended in 11 mL of freshly distilled THF under argon. 745 mg (2 eq, 2.84 mmol) of triphenylphosphine was then added, followed by 198 µL (1.5 eq, 2.13 mmol) of 4-pentyn-1-ol. The flask was then placed in an ice bath and stirred for 10 min. 391 µL (1.4 eq, 1.99 mmol) of diisopropylazodicarboxylate (DIAD) was then added drop-wise. The reaction was stirred, allowing it to return to room temperature, for 72 h at which point the reaction appeared done by TLC (EtOAc, KMnO_4_ stain). The solvent was removed under reduced pressure, giving a viscous oil that was then subjected to high vacuum for 1 h before being dissolved in the minimum amount of ethyl acetate. After purification by flash chromatography (50% EtOAc in hexanes), 350 mg (89% yield) of the product was obtained as a white powder. mp 107.6–108.6 °C; IR (KBr) 3270.68, 2964.05, 1727.91, 1604.48, 1444.42, 1371.14, 1270.86, 1112.73, 1045.23, 1008.59, 883.24, 788.74, 705.82, 592.04 cm^−1^; ^1^H-NMR (400 MHz, CDCl3) δ 7.8 (2H, s), 4.2 (2H, t, *J* = 6.1 Hz), 4.0 (6H, s), 2.4 (2H, t of d, *J* = 6.8 Hz), 2.02 (2H, p, *J* = 6.4 Hz), 1.96 (1H, t, *J* = 2.7 Hz); ^13^C-NMR (100 MHz, CDCl_3_) δ 166.8, 165.1, 149.7, 114.5, 82.5, 69.5, 67.1, 53.2, 27.5, 14.9; ESIMS *m*/*z* 300.1 ([M + Na]^+^, 100%), 278.1 ([M + H]^+^, 25%); HRESIMS calc for C_14_H_15_NO_5_Na^+^ 300.08424, found 300.08404.

*Dimethyl 4-(hex-5-yn-1-yloxy)pyridine-2,6-dicarboxylate* (**3b**). Following the general procedure above but employing 5-hexyn-1-ol afforded 180 mg (87% yield) of **3b** after flash chromatography as a white solid. ^1^H-NMR (400 MHz, CDCl_3_) δ 7.8 (s, 2H), 4.1 (2H, t, *J* = 6.3 Hz), 4.0 (6H, s), 2.3 (2H, t of d, *J* = 7.0 Hz), 1.9–2.0 (m, 3H), 1.7 (2H, p, *J* = 7.2 Hz); ^13^C-NMR (100 MHz, CDCl_3_) δ 166.9, 165.1, 149.7, 114.5, 83.5, 69.0, 68.4, 53.2, 27.6, 24.6, 18.0; ESIMS *m*/*z* 605.2 ([2M + Na]^+^, 100%), 314.1 ([M + Na]^+^, 25%), 292.1 ([M + H]^+^,10%); HRESIMS calc for C_15_H_18_NO_5_^+^ 292.11790, found 292.11800.

*Dimethyl 4-(hept-6-yn-1-yloxy)pyridine-2,6-dicarboxylate* (**3c**). Following the general procedure above but employing 6-heptyn-1-ol afforded 164 mg (87% yield) of **3c** after flash chromatography as a white solid. ^1^H-NMR (400 MHz, CDCl_3_) δ 7.8 (2H, s), 4.11 (2H, t, *J* = 6.4 Hz), 3.96 (6H, s), 2.20 (2H, p, *J* = 2.7 Hz), 1.9 (1H, t, *J* = 2.7 Hz), 1.8 (2H, p, *J* = 6.8 Hz), 1.6 (4H, m). ^13^C-NMR (100 MHz, CDCl_3_) δ 167.0,165.1, 149.6, 114.4, 84.0, 68.8, 68.6, 53.2, 28.2, 27.9, 24.9, 18.2; ESIMS *m/z* 633.2 ([2M + Na]^+^, 100%), 328.1 ([M + Na]^+^, 33%), 306.1 ([M + H]^+^, 15%); HRESIMS calc for C_16_H_19_NO_5_Na^+^ 328.11550, found 328.11580.

*Dimethyl 4-(non-8-yn-1-yloxy)pyridine-2,6-dicarboxylate* (**3d**). Following the general procedure above but employing 7-octyn-1-ol afforded 221 mg (quantitative yield) of **3d** after flash chromatography as a white powder. ^1^H-NMR (400 MHz, CDCl_3_) δ 7.7 (2H, s), 4.1 (2H, t, *J* = 6.3 Hz), 4.0 (6H, s), 2.1 (2H, t, *J* = 6.9 Hz), 1.9 (1H, t, *J* = 2.6Hz), 1.8 (2H, p, *J* = 7.0 Hz), 1.3–1.5 (8H, m); ^13^C-NMR (100 MHz, CDCl_3_) δ 167.0, 165.1, 149.6, 114.4, 84.4, 68.9, 68.2, 53.1, 28.60, 28.55, 28.4, 28.2, 25.6, 18.3; CIMS *m*/*z* 334 ([M + H]^+^,100%); HRCIMS for C_18_H_25_NO_5_^+^ 334.1654, found 134.1655.

Trimethyl Aluminum Amidation General Procedure: 4-(pent-4-yn-1-yloxy)-*N*2,*N*6-di(quinolin-3-yl)pyridine-2,6-dicarboxamide (**4a**). 320 mg (1 eq, 1.04 mmol) of **3a** and 357 mg (2.4 eq, 2.5 mmol) of 3-aminoquinoline were dissolved in 15 mL distilled 1,2-dichloroethane under argon. 4.5 mL (4.2 eq, 4.5 mmol) of 1.0 M trimethylaluminium in heptane was then added under increased argon flow resulting in a yellow solution. The flask was then fitted with an oven-dried condenser and placed in an oil bath at 94 °C and stirred at reflux for 90 min at which point the reaction appeared complete by TLC (EtOAc). The dark red solution was cooled to room temperature and quenched with 3 mL of methanol, resulting in the formation of a yellow gel. The gel was diluted and partially dissolved with additional methanol and chloroform, silica gel added, and the solvent removed under reduced pressure. The silica-adsorbed residue was partially purified through a silica column plug (EtOAc). Combining the fractions from the column gave 564 mg of the crude product after removal of the solvent under reduced pressure. The crude product was then suspended in 20 mL of methanol and stirred for 2 h before being filtered under reduced pressure and washed three times with methanol giving 487 mg of the product as a yellow solid. The filtrate was concentrated under reduced pressure, suspended in 3–5 mL of methanol and filtered again after 30 min, giving an additional 8 mg of the product as a yellow solid. The combined crops gave 495 mg (89% yield) of the product. mp = 235 °C; IR (KBr) 3126.18, 1675.34, 1606.65, 1543.35, 1492.28, 1376.56, 1340.15, 1225.99, 1045.45, 902.46, 750.69 cm^−1^; ^1^H-NMR (400 MHz, CDCl_3_, 4% MeOD) δ 11.1 (0.5H, s), 9.02–9.04 (4H, m), 7.971 (2H, s), 7.969 (2H, d, *J* = 8.0 Hz), 7.8 (2H, d, *J* = 8.1 Hz), 7.6 (2H, t, *J* = 7.6 Hz), 7.5 (2H, t, *J* = 7.2 Hz), 4.3 (2H, t, *J* = 6.1 Hz), 2.4 (2H, t of d, *J* = 6.9 Hz), 2.1 (2H, p, *J* = 6.5 Hz), 2.0 (1H, t, *J* = 2.6 Hz); ^13^C (100 MHz, CDCl_3_, 4% MeOD) δ 167.8, 162.8, 150.7, 144.6, 144.5, 131.6, 128.7, 128.2, 127.9, 127.3, 125.6, 125.5, 112.1, 82.6, 69.5, 67.2, 27.5, 14.9; ESIMS *m*/*z* 502.3 ([M + H]^+^, 100%); HRESIMS calc for C_30_H_24_N_5_O_3_^+^ 502.18737, found 502.1873.

*4-(Hex-5-yn-1-yloxy)-N2,N6-di(quinolin-3-yl)pyridine-2,6-dicarboxamide* (**4b**). Following the general procedure above but employing **3b** afforded 146 mg (64% yield) of **4b** as a yellow solid. mp = 204 °C; IR (KBr) 3293.32, 2941.37, 1667.96, 1604.95, 1537.16, 1491.22, 1468.93, 1423.99, 1374.2, 1337.97, 1278.61, 1226.4, 1176.96, 1144.2, 1098.44, 1034.38, 899.15, 780.04, 748.42, 661.48 cm^−1^; ^1^H-NMR (400 MHz, CDCl_3_ (2% MeOD)) δ 10.9 (1H, s), 9.0 (2H, s), 8.9 (2H, s), 7.92 (2H, d, *J* = 8.4 Hz), 7.88 (2H, s), 7.7 (2H, d, *J* = 8.1 Hz), 7.6 (2H, t, *J* = 7.5 Hz), 7.5 (2H, t, *J* = 7.4 Hz), 4.2 (2H, t, *J* = 6.3 Hz), 2.3 (2H, t, *J* = 6.9 Hz), 2.00 (1H, t, *J* = 2.6 Hz), 1.99 (2H, p, *J* = 6.3 Hz), 1.7 (2H, p, *J* = 7.1 Hz); ^13^C-NMR (100 MHz, CDCl_3_ (2% MeOD)) δ 167.8, 162.7, 162.6, 150.6, 144.64, 144.59, 131.5, 131.4, 128.6, 128.3, 128.1, 127.8, 127.3, 125.4, 125.3, 112.0, 83.6, 69.0, 68.5, 27.7, 24.7, 18.0 ; ESIMS *m*/*z* 516.2 ([M + H]^+^, 100%), 252.2 (M^2+^, 70%); HRESIMS calc for C_31_H_26_N_5_O_3_^+^ 516.20302, found 516.20327.

*4-(Hept-6-yn-1-yloxy)-N2,N6-di(quinolin-3-yl)pyridine-2,6-dicarboxamide* (**4c**). Following the general procedure above but employing **3c** afforded 104.6 mg (37% yield) of **4c** as a pale yellow solid. mp 199.8–202.1 °C (decomp); IR (KBr) = 3354.65, 3199.37, 1686.04, 1637.74, 1609.93, 1539.93, 1492.12, 1399.91, 1375.81, 1337.48, 1207.02, 1175.14, 1145.52, 1028.99, 901.20, 782.60, 752.33, 736.65, 656.88 cm^−1^; ^1^H-NMR (400 MHz, DMF) δ 11.4 (2H, s), 9.5 (2H, d, *J* = 2.5 Hz), 9.1 (2H, d, *J* = 2.4 Hz), 8.1 (4H, t, *J* = 9.3 Hz), 7.8 (2H, t, *J* = 7.6 Hz), 7.7 (2H, t, *J* = 7.5 Hz), 4.4 (2H, t, *J* = 6.5 Hz), 2.8 (1H, t, *J* = 2.6 Hz), 2.3 (2H, p, *J* = 4.0 Hz), 1.9 (2H, p, *J* = 7.0 Hz), 1.7 (4H, m); ^13^C-NMR (100 MHz, DMF) δ 168.8, 163.0, 151.6, 146.4, 145.9, 133.0, 129.6, 128.9, 128.8, 128.6, 127.8, 124.4, 112.1, 84.9, 70.9, 69.7, 28.8, 25.6, 18.5. ESIMS *m*/*z* 530.22([M + H]^+^, 100%); HRESIMS calc for C_32_H_28_N_5_O_3_^+^ 530.21867, found 530.21987.

*4-(Non-8-yn-1-yloxy)-N2,N6-di(quinolin-3-yl)pyridine-2,6-dicarboxamide* (**4d**). Following the general procedure above but employing **3d** afforded 134 mg (36% yield) of 4d as pale yellow crystals. mp 160.7–162.4 °C (decomp); IR (KBr) 3299.15, 2933.39, 2373.65, 2344.64, 1870.59, 1846.21, 1793.81, 1773.91, 1751.61, 1735.65, 1718.92, 1700.86, 1685.47, 1664.83, 1637.67, 1607.89, 1560.25, 1542.40, 1490.85, 1458.79, 1421.72, 1375.18, 1399.93, 1208.00, 1143.95, 1097.50, 1015.73, 897.82, 778.95, 746.47, 669.71, 473.38 cm^−1^; ^1^H-NMR (400 MHz, CDCl3) δ 10.2 (2H, s), 8.9 (2H, d, *J* = 2.5 Hz), 8.5 (2H, d, *J* = 2.3 Hz), 7.9 (2H, d, *J* = 8.3 Hz), 7.7 (2H, s), 7.53 (2H, t, *J* = 8.1 Hz), 7.50 (2H, t, *J* = 7.7 Hz), 7.4 (2H, t, *J* = 7.5 Hz), 4.0 (2H, t, *J* = 6.6 Hz), 2.2 (2H, t of d, *J* = 7.0 Hz), 2.0 (1H, t, *J* = 2.6 Hz), 1.8 (2H, p, *J* = 6.7 Hz), 1.3–1.6 (8H, m); ^13^C-NMR (100 MHz, CDCl3) δ 167.9, 162.3, 150.2, 145.2, 144.9, 130.8, 128.71, 128.67, 127.8, 127.6, 127.2, 125.2, 111.8, 84.6, 69.2, 68.3, 28.8, 28.7, 28.6, 28.3, 25.7, 18.44, 18.38. ESIMS *m*/*z* 558.25 ([M + H]^+^, 100%); HRESIMS calc for C_34_H_32_N_5_O_3_^+^ 558.24997, found 558.25046.

Copper-catylized coupling General Procedure: 4-(3-(1-(2-(1,3-dioxo-1*H*-benzo[de]isoquinolin-2(3*H*)-yl)ethyl)-1*H*-1,2,3-triazol-4-yl)propoxy)-*N2*,*N6*-di(quinolin-3-yl)pyridine-2,6-dicarboxamide (**8a**). The alkyne **3a** 20 mg (1 eq, 0.04 mmol) and 12.7 mg (1.2 eq, 0.048 mmol) of azide 5 [29] were dissolved in 3 mL DMF and placed under argon. A solution of 0.1 M copper triflate in water (440 µL, 0.044 mmol) was added, resulting in a dark green solution. A freshly prepared solution of 0.1 M sodium ascorbate in water (160 µL, 0.016 mmol) was added, and the flask was placed in an oil bath at 25 °C and stirred for 68 h until the reaction appeared complete by TLC (EtOAc). The DMF was removed under high vacuum and the residue partitioned between about 40 mL of chloroform and 40 mL of water. This mixture was stirred vigorously for 24 h before being transferred to a separation funnel. The aqueous layer was extracted twice more with smaller volumes of chloroform (about 10–20 mL) and the organic layers were combined, and the solvent removed. The residue was dissolved in a mixture of methanol and chloroform and absorbed to silica by removal of the solvent under reduced pressure. A mini column was then run (EtOAc up to 10% MeOH in EtOAc), giving 23 mg (76% yield) of 8a as a pale yellow solid. ^1^H-NMR (400 MHz, CDCl_3_ (4% MeOD)) δ 11.1 (exchanged, s), 9.1 (4H, s), 8.5 (2H, d, *J* = 7.6 Hz), 8.2 (2H, d, *J* = 7.8 Hz), 8.0 (4H, s), 7.9 (2H, d, *J* = 7.7 Hz), 7.6–7.7 (4H, m), 7.5–7.6 (3H, m), 4.72 (2H, s), 4.62 (2H, s), 4.2 (2H, s), 2.9 (2H, t, *J* = 7.3 Hz), 2.2 (2H, t, *J* = 6.1 Hz). ESIMS *m*/*z* 790.26 ([M + Na]^+^,100%), 768.28 ([M + H]^+^, 25%); HRESIMS calc for (C_44_H_33_N_9_O_5_Na)^+^ 790.24970, found 790.25100.

*4-(3-(1-(4-Benzoylbenzyl)-1H-1,2,3-triazol-4-yl)propoxy)-N2,N6-di(quinolin-3-yl)pyridine-2,6-dicarboxamide* (**9a**). Following the general procedure above but employing azide **6** afforded 5.5 mg (52% yield) of the **9a** as a pale yellow solid. ^1^H-NMR (400 MHz, CDCl_3_ (1.5% MeOD)) δ 10.8 (exchanged amide, s), 9.1 (2H, s), 9.0 (2H, s), 7.99 (2H, d, *J* = 7.2 Hz), 7.91 (2H, s), 7.8 (2H, d, *J* = 8.6 Hz), 7.68–7.72 (4H, m), 7.6 (2H, t, *J* = 7.3 Hz), 7.52 (2H, t, *J* = 7.3 Hz), 7.49 (1H, t, *J* = 1.3 Hz), 7.4 (2H, t, *J* = 7.6 Hz), 7.3 (2H, t, *J* = 8.3 Hz), 5.6 (2H, s), 4.2 (2H, t, *J* = 6.2 Hz), 2.9 (2H, t, *J* = 7.3 Hz), 2.3 (2H, p, *J* = 6.6 Hz). ESIMS *m*/*z* 739.3 ([M + H]^+^, 50%), 761.3 ([M + Na]^+^, 40%); HRESIMS calc for C_44_H_35_N_8_O_4_^+^ = 739.27760, found 739.27870.

*3-(4-(3-((2,6-Bis(quinolin-3-ylcarbamoyl)pyridin-4-yl)oxy)propyl)-1H-1,2,3-triazol-1-yl)propyl 9,10-dioxo-9,10-dihydroanthracene-2-carboxylate* (**10a**). Following the general procedure above but employing azide 7 afforded 7 mg of **10a** (21% yield). ^1^H-NMR (400 MHz, CDCl_3_) δ 10.3 (2H, s), 9.1 (2H, s), 8.8 (1H, s), 8.7 (2H, s), 8.2–8.3 (2H, m), 8.16–8.18 (1H, m), 7.93 (2H, d, *J* = 8.0 Hz), 7.8 (2H, s), 7.71–7.74 (2H, m), 7.69 (2H, d, *J* = 8.9 Hz), 7.6 (2H, t, *J* = 7.6 Hz), 7.45–7.49 (3H, m), 4.5 (2H, t, *J* = 6.7 Hz), 4.3 (2H, t, *J* = 6.7 Hz), 4.2 (2H, t, *J* = 5.8 Hz), 3.0 (2H, t, *J* = 7.2 Hz), 2.4 (2H, p, *J* = 6.4 Hz), 2.3 (2H, p, *J* = 6.7 Hz). ESIMS *m*/*z* 859.3 ([M + Na]^+^, 50%); HRESIMS calc for C_48_H_36_N_8_O_7_Na^+^ 859.25990, found 859.26200.

*4-(4-(1-(2-(1,3-Dioxo-1H-benzo[de]isoquinolin-2(3H)-yl)ethyl)-1H-1,2,3-triazol-4-yl)butoxy)-N2,N6-di(quinolin-3-yl)pyridine-2,6-dicarboxamide* (**8b**). Following the general procedure above but employing alkyne **4b** afforded 13.4 mg (44% yield) of **8b**. ^1^H-NMR (400 MHz, CDCl_3_ (20% MeOD)) δ 9.0 (2H, s), 8.9 (2H, s), 8.4 (2H, d, *J* = 7.0 Hz), 8.1 (2H, d, *J* = 8.0 Hz), 7.91 (2H, d, *J* = 8.5 Hz), 7.89 (2H, s), 7.8 (2H, d, *J* = 8.3 Hz), 7.64 (2H, t, *J* = 7.8 Hz), 7.58 (2H, t, *J* = 7.6 Hz), 7.48 (2H, t, *J* = 7.6 Hz), 7.45 (1H, s), 4.6 (2H, t, *J* = 6.3 Hz), 4.5 (2H, t, *J* = 6.0 Hz), 4.1 (2H, t, *J* = 6.3 Hz), 2.7 (2H, t, *J* = 7.5 Hz), 1.8 (4H, m). ESIMS *m*/*z* 804.3 ([M + Na]^+^, 100%); HRESIMS calc for C_45_H_35_N_9_O_5_Na^+^ 804.26530, found 804.26690.

*4-(4-(1-(4-Benzoylbenzyl)-1H-1,2,3-triazol-4-yl)butoxy)-N2,N6-di(quinolin-3-yl)pyridine-2,6-dicarboxamide* (**9b**). Following the general procedure above but employing alkyne **4b** and azide **6** afforded 27.9 mg (95% yield) of **9b**. ^1^H-NMR (400 MHZ, CDCl_3_) δ 10.8 (s, exchanged), 9.1 (2H, s), 9.0 (2H, s), 8.01 (2H, d, *J* = 8.8 Hz), 7.93 (2H, s), 7.8 (2H, d, *J* = 8.4 Hz), 7.71–7.73 (4H, m), 7.6 (2H, t, *J* = 7.5 Hz), 7.5–7.6 (3H, m), 7.42 (2H, t, *J* = 7.7 Hz), 7.35 (1H, s), 7.3 (2H, d, *J* = 8.0 Hz), 5.6 (2H, s), 4.2 (2H, t, *J* = 5.4 Hz), 2.8 (2H, t, *J* = 6.8 Hz), 1.90–1.91 (4H, m). ESIMS *m*/*z* 775.28 ([M + Na]^+^, 100%), 753.29 ([M + H]^+^, 33%); HRESIMS calc for (C_45_H_36_N_8_O_4_Na)^+^ 775.27520, found 775.27540.

*4-((5-(1-(4-Benzoylbenzyl)-1H-1,2,3-triazol-4-yl)pentyl)oxy)-N2,N6-di(quinolin-3-yl)pyridine-2,6-dicarboxamide* (**9c**). Following the general procedure above but employing alkyne **4c** and azide **6** afforded 34.6 mg (88% yield) of **9c** as an off-white solid mp 125 °C; IR (KBr) 3128.25, 2935.02, 2385.38, 1794.12, 1774.05, 1735.74, 1719.09, 1701.17, 1686.19, 1655.31, 1637.99, 1607.92, 1578.00, 1560.39, 1543.06, 1509.50, 1490.94, 1459.13, 1400.02, 1099.73, 750.87, 612.45 cm^−1^; ^1^H-NMR (400 MHZ, CDCl_3_) δ 10.2 (2H, s), 9.1 (2H, d, *J* = 2.5 Hz), 8.8 (2H, d, *J* = 2.3 Hz), 8.0 (2H, d, *J* = 9.3 Hz), 7.9 (2H, s), 7.8 (2H, d, *J* = 8.2 Hz), 7.69–7.72 (4H, m), 7.6 (2H, t, *J* = 7.7 Hz), 7.5–7.6 (3H, m), 7.4 (2H, t, *J* = 7.0 Hz), 7.26–7.28 (3H, m), 5.6 (2H, s), 4.1 (2H, t, *J* = 6.4 Hz), 2.8 (2H, t, *J* = 7.5 Hz), 1.9 (2H, p, *J* = 6.9 Hz), 1.8 (2H, p, *J* = 7.6 Hz), 1.5 (2H, p, *J* = 7.6 Hz); ESIMS *m*/*z* 789.29 ([M + Na]^+^, 100%), 767.31 ([M + H]^+^, 70%); HRESIMS calc for (C_46_H_38_N_8_O_4_Na)^+^ 789.29080, found 789.29120. 

*4-((7-(1-(4-Benzoylbenzyl)-1H-1,2,3-triazol-4-yl)heptyl)oxy)-N2,N6-di(quinolin-3-yl)pyridine-2,6-dicarboxamide* (**9d**). Following the general procedure above but employing alkyne **4d** and azide **6** afforded 20.9 mg (49% yield) of **9d** as a white powder. ^1^H-NMR (400 MHz, CDCl_3_) δ 10.1 (2H, s), 9.1 (2H, d, *J* = 2.9 Hz), 8.8 (2H, d, *J* = 2.6 Hz), 8.0 (2H, d, *J* = 8.6 Hz), 7.9 (2H, s), 7.7–7.8 (6H, m), 7.62 (2H, t, *J* = 7.7 Hz), 7.5–7.56 (3H, m), 7.4 (2H, t, *J* = 7.2 Hz), 7.2–7.3 (3H, m), 5.5 (2H, s), 4.2 (2H, t, *J* = 6.4 Hz), 2.7 (2H, t, *J* = 7.7 Hz), 1.8 (2H, p, *J* = 7.0 Hz), 1.7 (2H, p, *J* = 7.5 Hz), 1.5 (2H, p, *J* = 6.4 Hz), 1.4–1.47 (4H, m). ESIMS *m*/*z* 795.3 ([M + H]^+^, 50%), 817.3 ([M + Na]^+^, 100%), 1612.7 ([2M + Na]^+^, 30%); HRESIMS calc for C_48_H_42_N_8_O_4_Na^+^ 817.32210, found 817.32210.

*3-(4-(4-((2,6-Bis(quinolin-3-ylcarbamoyl)pyridin-4-yl)oxy)butyl)-1H-1,2,3-triazol-1-yl)propyl 9,10-dioxo-9,10-dihydroanthracene-2-carboxylate* (**10b**). Following the general procedure above but employing alkyne **4b** and azide 7 afforded 9.7 mg of **10b** as a white solid (29% yield). ^1^H-NMR (400 MHz, CDCl_3_ (5% MeOD)) δ 9.0 (4H, s), 8.8 (1H, s), 8.2–8.4 (4H, m), 7.99 (2H, d, *J* = 8.2 Hz), 7.95 (2H, s), 7.8 (2H, d, *J* = 8.4 Hz), 7.75–7.77 (2H, m), 7.6 (2H, t, *J* = 7.3 Hz), 7.54 (2H, t, *J* = 7.5 Hz), 7.45 (1H, s), 4.5 (2H, t, *J* = 7.0 Hz), 4.4 (2H, t, *J* = 6.1 Hz), 4.2 (2H, t, *J* = 6.4 Hz), 2.8 (2H, t, *J* = 6.7 Hz), 2.4 (2H, p, *J* = 6.4 Hz), 1.9 (4H, m); ESIMS *m*/*z* 873.3 ([M + Na]^+^, 50%); HRESIMS calc for C_49_H_38_N_8_O_7_Na^+^ 873.27560, found 873.27450.

Methylation General Procedure: 3,3'-((4-(4-(1-(2-(1,3-dioxo-1*H*-benzo[de]isoquinolin-2(3*H*)-yl)ethyl)-3-methyl-1*H*-1,2,3-triazol-3-ium-4-yl)butoxy)pyridine-2,6-dicarbonyl)bis(azanediyl))bis(1-methylquinolin-1-ium) trifluoromethanesulfonate (**11b**). 13.4 mg (1 eq, 0.016 mmol) of 8b was dissolved in 3 mL of 3.8% methanol in chloroform and placed under argon. The flask was placed in an ice bath and 10 µL (5.6 eq, 0.09 mmol) of methyl triflate was added slowly. The solution was then stirred for 4 h, allowing the bath to return to room temperature. A significant amount of precipitate began to form after 2 h. This precipitate was isolated by filtration under reduced pressure and washed twice with small (1 mL) portions of chloroform, giving 13.4 mg (66% yield). ^1^H-NMR (400 MHz, *d*_6_*-*DMSO) δ 11.9 (2H, s), 10.0 (2H, s), 9.6 (2H, s), 8.9–9.0 (2H, m), 8.4–8.6 (5H, m), 8.3 (2H, t, *J* = 8.0 Hz), 8.05–8.11 (2H, m), 7.92 (2H, s), 7.86 (2H, t, *J* = 7.5 Hz), 7.7–7.8 (2H, m), 5.0 (2H, t, *J* = 5.5 Hz), 4.8 (6H, s), 4.6 (2H, t, *J* = 5.7 Hz), 4.3 (2H, s), 4.2 (3H, s), 3.0 (2H, t, *J* = 6.6 Hz), 1.8 (4H, m). MALDIMS *m*/*z* 1124 ([M − OTf], 100%); HRMALDIMS calc for C_50_H_44_N_9_O_11_F_6_S_2_^+^ 1124.25004, found 1124.24914. 

*3,3'-((4-(3-(1-(2-(1,3-Dioxo-1H-benzo[de]isoquinolin-2(3H)-yl)ethyl)-3-methyl-1H-1,2,3-triazol-3-ium-4-yl)propoxy)pyridine-2,6-dicarbonyl)bis(azanediyl))bis(1-methylquinolin-1-ium) trifluoromethanesulfonate* (**11a**). Following the general procedure above but starting with the triazole 8a afforded 3.5 mg (21% yield) of **11a** ^1^H-NMR (400 MHz, *d*_6_*-*DMSO) δ 10.1 (2H, s), 9.6 (2H, s), 9.0 (2H, s), 8.6 (2H, t, *J* = 8.1 Hz), 8.4 (3H, m), 8.3 (2H, t, *J* = 8.0 Hz), 8.07–8.11 (3H, m), 7.93–7.97 (2H, m), 7.7–7.8 (4H, m), 5.0 (2H, s), 4.8 (6H, s), 4.6 (2H, s), 4.4 (2H, s), 4.2 (3H, s), 3.1 (2H, t, *J* = 7.2 Hz), 2.2 (2H , s). MALDIMS *m*/*z* 1110.1 ([M − OTf]^+^, 8%). HRMALDIMS calc for C_49_H_42_N_9_O_11_F_6_S_2_^+^ 1110.23439, found 1110.2335.

*3,3'-((4-(3-(1-(4-Benzoylbenzyl)-3-methyl-1H-1,2,3-triazol-3-ium-4-yl)propoxy)pyridine-2,6-dicarbonyl)bis(azanediyl))bis(1-methylquinolin-1-ium) trifluoromethanesulfonate* (**12a**). Following the general procedure above but starting with the triazole **9a** afforded 8.7 mg (50% yield) of **12a**. ^1^H-NMR (600 MHz, *d*_6_-DMSO) δ 11.8 (2H, s), 10.0 (2H, s), 9.6 (2H, s), 9.0 (1H, s), 8.6 (2H, d, *J* = 9.0 Hz), 8.5 (2H, d, *J* = 7.8 Hz), 8.3 (2H, t, *J* = 7.9 Hz), 8.1 (2H, t, *J* = 7.8 Hz), 8.0 (2H, s), 7.8 (2H, d, *J* = 6.6 Hz), 7.6–7.7 (5H, m), 7.5 (2H, t, *J* = 6.8 Hz), 6.0 (2H, s), 4.8 (6H, s), 4.5 (2H, t, *J* = 6.0 Hz), 4.3 (3H, s), 3.1 (2H, t, *J* = 7.8 Hz), 2.3 (2H, p, *J* = 6.9 Hz ). ESIMS *m*/*z* 261 (M^3+^, 80%); HRESIMS calc for C_47_H_43_N_8_O_4_^3+^ 261.11300, found 261.11310.

*3,3'-((4-(4-(1-(4-Benzoylbenzyl)-3-methyl-1H-1,2,3-triazol-3-ium-4-yl)butoxy)pyridine-2,6-dicarbonyl)bis(azanediyl))bis(1-methylquinolin-1-ium) trifluoromethanesulfonate* (**12b**). Following the general procedure above but starting with the triazole **9b** afforded 5.7 mg (35% yield) of 12b ^1^H-NMR (400 MHz, *d*_6_*-*DMSO) δ 10.0 (2H, s), 9.6 (2H, s), 8.9 (2H, s), 8.6 (4H, t, *J* = 7.2 Hz), 8.3 (2H, t, *J* = 8.0 Hz), 8.1 (2H, t, *J* = 7.8 Hz), 8.0 (2H, s), 7.8 (2H, d, *J* = 8.2 Hz), 7.62–7.7 (4H, m), 7.56 (2H, t, *J* = 8.0 Hz), 6.0 (2H, s), 4.8 (6H, s), 4.4 (2H, m), 4.2 (3H, s), 3.0 (2H, t, *J* = 7.0 Hz), 1.9 (4H, m); MALDIMS *m*/*z* 1095 ([M − OTf], 100%); HRMALDIMS calc for C_50_H_45_N_8_O_10_F_6_S_2_^+^ 1095.25988, found 1095.258.

*3,3'-((4-((5-(1-(4-Benzoylbenzyl)-3-methyl-1H-1,2,3-triazol-3-ium-4-yl)pentyl)oxy)pyridine-2,6-dicarbonyl)bis(azanediyl))bis(1-methylquinolin-1-ium) trifluoromethanesulfonate* (**12c**). Following the general procedure above but starting with the triazole **9c** afforded 10.1 mg (53% yield) of **12c** as a white powder. mp 211 °C; IR (KBr) = 3122.83, 2372.10, 2344.75, 1774.14, 1735.85, 1719.20, 1701.46, 1686.39, 1655.16, 1637.81, 1560.41, 1543.41, 1475.80, 1458.82, 1399.46, 1275.61, 1156.44, 1030.19, 637.63 cm^−^^1^; ^1^H-NMR (400 MHz, *d*_7_*-*DMF) δ 11.9 (2H, s), 10.3 (2H, d, *J* = 2.6 Hz), 9.8 (2H, s), 9.1 (1H, s), 8.7 (2H, d, *J* = 9.2 Hz), 8.6 (2H, d, *J* = 8.4 Hz), 8.4 (2H, s), 8.3 (2H, t, *J* = 8.0 Hz), 8.2 (2H, t, *J* = 7.5 Hz), 8.02–8.05 (2H + DMF, m), 7.9 (2H, d, *J* = 8.1 Hz), 7.7–7.8 (5H, m), 7.6 (2H, t, *J* = 7.6 Hz), 6.2 (2H, s), 5.0 (6H, s), 4.5 (3H, s), 3.1 (2H, t, *J* = 7.6 Hz), 1.9–2.0 (4H, m), 1.7 (2H, p, *J* = 7.7 Hz); ESIMS *m*/*z* 270.5 (M^3+^, 1.5%), 398.2 ([M − Me]^2+^, 100%); HRESIMS calc for C_49_H_47_N_8_O_4_^3+^ = 270.45679, found 270.45621; HRESIMS calc for C_48_H_44_N_8_O_4_^2+^ 398.17373, found 398.17423.

*3,3'-((4-((7-(1-(4-Benzoylbenzyl)-3-methyl-1H-1,2,3-triazol-3-ium-4-yl)heptyl)oxy)pyridine-2,6-dicarbonyl)bis(azanediyl))bis(1-methylquinolin-1-ium) trifluoromethanesulfonate* (**12d**). Following the general procedure above but starting with the triazole **9d** afforded 2.5 mg (24% yield) of **12d** as a white powder. ^1^H-NMR (400 MHz, *d*_7_*-*DMF) δ 12.0 (2H, s), 10.3 (2H, s), 9.8 (2H, s), 8.7 (2H, d, *J* = 8.6 Hz), 8.6 (2H, d, *J* = 8.6 Hz), 8.3 (2H, t, *J* = 8.6 Hz), 8.2 (2H, t, *J* = 8.1 Hz), 8.1 (2H, s), 7.9 (2H, d, *J* = 6.7 Hz), 7.7–7.8 (4H, m), 7.6 (2H, t, *J* = 7.7 Hz), 6.2 (2H, s), 5.0 (6H, s), 4.4 (3H, s), 3.1 (2H, t, *J* = 8.4 Hz), 1.8–1.9 (4H, m), 1.5–1.6 (6H, m). MALDIMS *m*/*z* 1137.0 ([M − OTf]^+^, 15%); HRMS calc for C_53_H_51_N_8_O_10_F_6_S_2_ 1137.30683, found 1137.3037.

*3,3'-((4-(3-(1-(3-((9,10-Dioxo-9,10-dihydroanthracene-2-carbonyl)oxy)propyl)-3-methyl-1H-1,2,3-triazol-3-ium-4-yl)propoxy)pyridine-2,6-dicarbonyl)bis(azanediyl))bis(1-methylquinolin-1-ium) fluoromethanesulfonate* (**13a**). Following the general procedure above but starting with the triazole **10a** afforded 7.4 mg (66% yield) of **13a**. ^1^H-NMR (400 MHz, *d*_6_*-*DMSO) δ 10.0 (2H, s), 9.6 (2H, s), 9.0 (2H, s), 8.6 (2H, s), 8.5 (2H, d, *J* = 8.3 Hz), 8.30–8.34 (2H, m), 8.2 (2H, t, *J* = 7.8 Hz), 8.18–8.23 (2H, m), 8.1 (2H, t, *J* = 7.4 Hz), 7.92–7.94 (2H, m), 7.90 (2H, s), 4.83 (2H, t, *J* = 6.5 Hz), 4.78 (6H, s), 4.5 (2H, t, *J* = 5.9 Hz), 4.4 (2H, t, *J* = 6.2 Hz), 4.2 (3H, s), 3.1 (2H, t, *J* = 7.4 Hz), 2.18–2.20 (2H, m). ESIMS *m*/*z* 293.8 (M^3+^, 60%); HRESIMS calc for C_51_H_45_N_8_O_7_^3+^ 293.77980, found 293.78040.

*3,3'-((4-(4-(1-(3-((9,10-Dioxo-9,10-dihydroanthracene-2-carbonyl)oxy)propyl)-3-methyl-1H-1,2,3-triazol-3-ium-4-yl)butoxy)pyridine-2,6-dicarbonyl)bis(azanediyl))bis(1-methylquinolin-1-ium) trifluoromethanesulfonate* (**13b**). Following the general procedure above but starting with the triazole **10b** afforded 3.5 mg (22% yield) of **13b**. ^1^H-NMR (400 MHz, *d*_7_*-*DMF) δ 11.9 (2H, s), 10.2 (2H, s), 9.8 (2H, s), 9.1 (2H, s), 8.69–8.72 (3H, m), 8.6 (2H, d, *J* = 8.3 Hz), 8.35–8.41 (4H, m), 8.2–8.3 (4H, m), 8.1–8.2 (3H, m), 5.1 (2H, t, *J* = 6.7 Hz), 5.0 (6H, s), 4.6 (2H, t, *J* = 5.7 Hz), 4.5 (2H, t, *J* = 5.5 Hz), 4.4 (3H, s), 3.2 (2H, m), 2.1 (6H, m). MALDIMS *m*/*z* 1193 ([M − OTf]^+^, 100%). HRMALDIMS calc for C_54_H_47_N_8_O_13_F_6_S_2_^+^ 1193.26082, found 1193.2609. 

*N*-(2-(Dimethylamino)ethyl)-9,10-dioxo-9,10-dihydroanthracene-2-carboxamide (**14**). 100 mg (1 eq, 0.37 mmol) of 9,10-dioxo-9,10-dihydroanthracene-2-carbonyl chloride was suspended in 4 mL of dichloromethane. A solution of 56 µL *N*,*N*-dimethylethylenediamine in 6 mL dichloromethane was slowly added by addition funnel over 35 min. The mixture was then stirred at 35 °C for 2 h, when an additional 50 µL of diamine were added dropwise and the mixture stirred for an additional hour, at which point the reaction appeared to be complete by TLC (75% EtOAc in Hexanes). The precipitate was isolated by filtration under reduced pressure and was washed 3X with dichloromethane, giving 46.7 mg of starting material (by TLC). The filtrate was concentrated under reduced pressure and the residue partitioned between dichloromethane and water. The aqueous layer was extracted an additional two times with dichloromethane, and the combined organic layers washed once with 2.3 M NaOH and once with brine before being dried over sodium sulfate. Removal of the solvent under reduced pressure gave 54 mg (46% yield) of product as a yellow solid. ^1^H-NMR (400 MHz, CDCl_3_) δ 8.5 (1H, s), 8.2–8.3 (4H, m), 7.6–7.7 (2H, m), 7.2 (1H, s), 3.5 (2H, q, *J* = 5.6 Hz), 2.5 (2H, t, *J* = 6.0 Hz), 2.3 (6H, s); ^13^C-NMR (100 MHz, CDCl_3_) δ 182.43, 182.36, 165.6, 139.6, 134.9, 134.31, 134.28, 133.28, 133.25, 133.0, 127.7, 127.3 125.2, 57.6, 45.1, 37.5.

### 3.3. G-Quadruplex DNA Photocleavage Assays

The photocleavage experiments were adapted from the published photocleavage assay [21]. Briefly, solutions containing a single library compound and 100 nmol FRET pair-labeled DNA or blank controls in potassium cacodylate buffer were prepared and then added in triplicate to a 384-well plate (20 µL reaction volume) and irradiated in a Luzchem photoreactor for 30 min before being diluted to 95 µL with dissociation buffer (large excess calf thymus DNA in potassium cacodylate buffer). The plate was then sealed and heated in an oven at 85 °C for 30 min before being slowly cooled to room temperature overnight. After spinning down condensation in the plates, triplicate wells from the plate were combined, then split into two samples, one of which was treated with piperidine and heat while the other remained untreated. These samples were dried down under vacuum and resuspended in formamide denaturing buffer prior to being subjected to 20% denaturing PAGE at 8 W (constant current). Gels were visualized using fluorescein fluorescence on a Typhoon Trio gel reader. Cleavage bands within a lane were normalized against total lane signal and control lane signals subtracted so that the relative cleavage in each lane could be determined to give % cleavage of the DNA strand above background. Band intensities were quantified using GelQuant. NET provided by biochemlabsolutions.com.

Percent Cleavage = ((C_x_/Tot_x_ − FC_0_)/(1 − FC_0_)) × 100 (1)

where C_x_ is the sum of the cleavage band intensities, Tot_x_ is the total band intensities in a lane, and FC_0_ is the ratio of cleavage band intensities to total band intensities in a control lane loaded with the untreated control.

### 3.4. G-Quadruplex DNA Melting Assays

200 nM of FRET pair-labeled DNA in 5 mM potassium cacodylate buffer containing 0.25 mM disodium EDTA was first precycled (heated to 100 °C, then cooled slowly back to 10 °C) three times prior to being run to help improve reproducibility between replicate DNA melts. Precycled DNA samples were then treated with 0 nM–1 µM compound and incubated for 15 min prior to being heated in a Varian Eclipse fluorimeter from 10 °C to 80 °C at 1 °C/min and from 80 °C to 100 °C at 0.5 °C /min before resting at 100 °C for 2 min and then cooling to 10 °C at 1 °C/min. The melting traces followed the fluorescence signal of fluorescein (FAM) and data was fit to the logistic equation below through nonlinear regression using SciDavis in order to determine melting points.

I = I_0_ + (I_f_ − I_0_)/(1 + exp(− s × (T − T_m_))) (2)

where I is fluorescence intensity, I_0_ is initial fluorescence intensity, I_f_ is final fluorescence intensity, s is the signal increase rate (a scaling factor), T is temperature, and T_m_ is the melting temperature.

## 4. Conclusions 

The strategy of combining a “targeting” moiety with a “warhead” moiety through “click” chemistry still shows some promise, although the levels of both photocleavage and binding were less than originally expected. This could be due in part to the strong dependence of binding on the length of the linker used as well as the non-ideal photocleavage moiety positioning due to the charge introduced to the linker. Preparation of another library that does not incorporate this charge while continuing the exploration of linker length could improve both the binding and photocleavage activity, giving analogues that are better suited as photoactive probes for G4 DNA.
